# Trends in Mortality from Cerebrovascular and Hypertensive Diseases in Brazil Between 1980 and 2012

**DOI:** 10.5935/abc.20160092

**Published:** 2016-07

**Authors:** Paolo Blanco Villela, Carlos Henrique Klein, Gláucia Maria Moraes de Oliveira

**Affiliations:** 1 Universidade Federal do Rio de Janeiro, Rio de Janeiro, RJ - Brazil; 2 Escola Nacional de Saúde Pública, Rio de Janeiro, RJ - Brazil

**Keywords:** Cardiovascular Diseases / mortality, Hypertension / mortality, Epidemiology, Mortality

## Abstract

**Background:**

Cerebrovascular and hypertensive diseases are among the main causes of death worldwide. However, there are limited data about the trends of these diseases over the years.

**Objective:**

To evaluate the temporal trends in mortality rates and proportional mortality from cerebrovascular and hypertensive diseases according to sex and age in Brazil between 1980 and 2012.

**Methods:**

We evaluated the underlying causes of death between 1980 and 2012 in both sexes and by age groups for circulatory diseases (CD), cerebrovascular diseases (CBVD), and hypertensive diseases (HD). We also evaluated death due to all causes (AC), external causes (EC), and ill-defined causes of death (IDCD). Data on deaths and population were obtained from the Department of Information Technology of the Unified Health System (Departamento de Informática do Sistema Único de Saúde, DATASUS/MS). We estimated crude and standardized annual mortality rates per 100,000 inhabitants and percentages of proportional mortality rates.

**Results:**

With the exception of EC, the mortality rates per 100,000 inhabitants of all other diseases increased with age. The proportional mortality of CD, CBVD, and HD increased up to the age range of 60-69 years in men and 70-79 years in women, and reached a plateau in both sexes after that. The standardized rates of CD and CBVD declined in both sexes. However, the HD rates showed the opposite trend and increased mildly during the study period.

**Conclusion:**

Despite the decline in standardized mortality rates due to CD and CBVD, there was an increase in deaths due to HD, which could be related to factors associated with the completion of the death certificates, decline in IDCD rates, and increase in the prevalence of hypertension.

## Introduction

Chronic noncommunicable diseases are the main causes of death worldwide. Only in 2010, they accounted for almost 35 million deaths, 30% more of those that occurred in 1990.^1^ Among them, ischemic heart diseases (IHD) and cerebrovascular diseases (CBVD), the main representatives of all circulatory diseases (CD), accounted in 2012 for 7.4 and 6.7 million deaths, respectively.^2^ This global data is also observed in Brazil, where according to the Department of Information Technology of the Unified Health System (*Departamento de Informática do Sistema Único de Saúde*, DATASUS),^3^ 28.2% of the underlying causes of death in 2012 were attributed to CD (333.295). Among these deaths due to CD, 31.1% were due to IHD, 30.1% to CBVD, and 13.6% to hypertensive diseases (HD).

In order to improve the treatment of a disease and reduce the number of deaths it is associated with, it is fundamental to understand the long-term trends of this disease. Trends in mortality from CBVD have been assessed in Europe and other regions of the world.^4,5^ However, there is limited information in Brazil on the distribution, trends, and mortality data of CBVD. In addition, data published about these diseases only show their trends in some states^6-8^ or related to a period range below 10 years.^9^

These data are of such importance that similar information has allowed a project entitled Global Burden of Disease^10^ to assess the impact of noncommunicable diseases in several countries between 1990 and 2013.^10^ With these data, Roth et al.^11^ evaluated the mortality from cardiovascular diseases and concluded that their main determinants were related to growth and aging of the population, with little association with the gross national product (GNP) *per capita*.

Considering that there is a limited dissemination of available data on a national level regarding the long-term course of CBVD and HD, this study aimed at evaluating the temporal trends in mortality rates and proportional mortality associated with these diseases and according to sex and age in Brazil from 1980 to 2012.

## Methods

This was an ecological and descriptive study of historical series of death registries that occurred in Brazil between 1980 and 2012 and included all age ranges and both sexes.

Data on the underlying causes of deaths were obtained from the DATASUS website.^3^ The original files of death certificates are in a DBC format and were converted into the XLS format with the program Tabwin.^12^ The deaths were classified according to the following groups of causes: all causes (AC); external causes (EC; ICD-9 chapter XVII and ICD-10 chapter XX), ill-defined causes (IDCD; ICD-9 chapter XVI and ICD-10 chapter XVIII), CD (ICD-9 chapter VII and ICD-10 chapter IX), CBVD (ICD-9 codes 430-438 and ICD-10 codes I60-I69), and HD (ICD-9 codes 401-405 and ICD-10 codes I10-I15). ICD-9 codes were used between 1980 and 1995,^13^ and ICD-10 codes were used from 1996 to 2012.^14^

Age was stratified into the following groups: up to 29 years, 30-39 years, 40-49 years, 50-59 years, 60-69 years, 70-79 years, and 80 years or more. We created three periods of 10 years: 1980 to 1989, 1990 to 1999, and 2000 to 2009, in addition to a period of 3 years, from 2010 to 2012. We estimated crude and standardized annual mortality rates per 100,000 inhabitants using the direct method,^15^ and used the age distribution of the Brazilian population from 1980 to 2012 as a standard. We also estimated proportional mortality coefficients, which were reported in percentages.

The population data were obtained from the DATASUS website,^3^ which shows the age distributions of the surveyed populations in the years 1980, 1991, 2000, and 2010. We also obtained from the Brazilian Institute of Geography and Statistics (*Instituto Brasileiro de Geografia e Estatística*, IBGE) data related to the 1996 population count. The populations in the years between censuses were estimated by arithmetic progression in segments between each census or population count for each age group.

We constructed Cartesian graphs of mortality rates by groups of underlying cause of death by periods of time and according to sex. We used the program Microsoft Excel®^16^ to analyze the data.

## Results

Tables 1 and 2 present the annual mortality rate per 100,000 inhabitants, the proportional mortality, and the sex ratios according to groups of selected causes, including AC of death, according to age ranges from 1980 to 2012.

**Table 1:** Mortality rates per groups of selected causes and sex, per 100 thousand inhabitants, and rate ratios between sexes, according to age groups – Brazil, from 1980 to 2012.

Cause	All causes			External Causes*			Ill-defined Causes**			CD***			CBVD****			HD*****		
	Mort./100,000		Ratio	Mort./100,000		Ratio	Mort./100,000		Ratio	Mort./100,000		Ratio	Mort./100,000		Ratio	Mort./100,000		Ratio
Age group	Men	Women	M/F	Men	Women	M/F	Men	Women	M/F	Men	Women	M/F	Men	Women	M/F	Men	Women	M/F
Up to 29 years	249.9	141.5	1.8	92.2	17.2	5.4	30.5	23.0	1.3	6.2	5.0	1.2	1.4	1.3	1.1	0.3	0.3	1.0
30 to 39 years	370.5	142.2	2.6	171.2	22.4	7.6	36.2	16.2	2.2	42.2	28.2	1.5	12.0	10.9	1.1	3.3	2.8	1.2
40 to 49 years	623.9	303.4	2.1	151.1	23.4	6.5	72.4	34.4	2.1	139.5	87.9	1.6	41.1	34.8	1.2	11.4	9.4	1.2
50 to 59 years	1224.2	660.7	1.9	138.1	25.8	5.4	142.4	72.4	2.0	388.4	221.3	1.8	114.6	78.1	1.5	32.4	24.3	1.3
60 to 69 years	2442.6	1458.2	1.7	131.3	34.4	3.8	297.8	169.6	1.8	895.6	546.7	1.6	276.4	177.1	1.6	73.2	58.3	1.3
70 to 79 years	5328.6	3674.4	1.5	163.9	69.6	2.4	773.2	504.2	1.5	2011.3	1473.2	1.4	677.9	496.6	1.4	164.6	149.9	1.1
80 years or more	12222.1	10542.4	1.2	289.1	213.9	1.4	2182.9	1700.0	1.3	4401.2	4232.1	1.0	1479.6	1388.7	1.1	416.5	461.5	0.9
Crude******	683.6	477.7	1.4	117.8	23.8	4.9	89.5	66.6	1.3	172.5	148.7	1.2	54.5	50.1	1.1	14.5	15.7	0.9
Standardized*******	724.4	447.4	1.6	118.7	23.3	5.1	96.0	62.1	1.5	188.0	136.2	1.4	59.6	46.0	1.3	15.8	14.3	1.1

* External cause: ICD-9 chapter XVII and ICD-10 chapter XX;

** Ill-defined cause: ICD-9 chapter XVI and ICD-10 chapter XVIII;

*** Circulatory disease (CD): ICD-9 chapter VII and ICD-10 chapter IX;

**** Cerebrovascular disease (CBVD): ICD-9 codes 430–438 and ICD-10 codes I60–I69;

***** Hypertensive disease (HD): ICD-9 codes 401–405 and ICD-10 codes I10–I15;

****** Overall rate (all ages);

******* Standardized rate by age group (standard: overall Brazilian population between 1980 and 2012)

**Table 2:** Percentages of proportional mortality per groups of selected causes and sex, and sex ratios, according to age groups – Brazil, from 1980 to 2012.

Cause	External Causes*			Ill-defined Causes**			CD***			CBVD****			HD*****		
	Prop. Mort. (%)		Ratio	Prop. Mort. (%)		Ratio	Prop. Mort. (%)		Ratio	Prop. Mort. (%)		Ratio	Prop. Mort. (%)		Ratio
Age group	Men	Women	M/F	Men	Women	M/F	Men	Women	M/F	Men	Women	M/F	Men	Women	M/F
Up to 29 years	36,9	12,2	3,0	12,2	16,3	0,7	2,5	3,6	0,7	0,6	0,9	0,6	0,1	0,2	0,6
30 to 39 years	46,2	15,8	2,9	9,8	11,4	0,9	11,4	19,8	0,6	3,2	7,7	0,4	0,9	2,0	0,5
40 to 49 years	24,2	7,7	3,1	11,6	11,3	1,0	22,4	29,0	0,8	6,6	11,5	0,6	1,8	3,1	0,6
50 to 59 years	11,3	3,9	2,9	11,6	11,0	1,1	31,7	33,5	0,9	9,4	11,8	0,8	2,6	3,7	0,7
60 to 69 years	5,4	2,4	2,3	12,2	11,6	1,0	36,7	37,5	1,0	11,3	12,1	0,9	3,0	4,0	0,7
70 to 79 years	3,1	1,9	1,6	14,5	13,8	1,0	37,7	40,4	0,9	12,7	13,6	0,9	3,1	4,1	0,8
80 years or more	2,4	2,0	1,2	17,9	16,1	1,1	36,0	40,1	0,9	12,1	13,2	0,9	3,4	4,4	0,8
Crude******	17,2	5,0	3,5	13,1	13,9	0,9	25,2	31,1	0,8	8,0	10,5	0,8	2,1	3,3	0,6
Standardized*******	16,4	5,2	3,1	13,3	13,9	1,0	26,0	30,4	0,9	8,2	10,3	0,8	2,2	3,2	0,7

* External cause: ICD-9 chapter XVII and ICD-10 chapter XX;

** Ill-defined cause: ICD-9 chapter XVI and ICD-10 chapter XVIII;

*** Circulatory disease (CD): ICD-9 chapter VII and ICD-10 chapter IX;

**** Cerebrovascular disease (CBVD): ICD-9 codes 430–438 and ICD-10 codes I60–I69;

***** Hypertensive disease (HD): ICD-9 codes 401–405 and ICD-10 codes I10–I15;

****** Overall proportional mortality (all ages);

******* Ratio between the specific rate and all standardized causes (by age groups. Standard: overall Brazilian population from 1980 to 2012)

With the exception of the deaths from EC, the mortality rates per 100,000 inhabitants for all other causes of death increased sharply with the advance of age. The more pronounced increases occurred in the rates of CD and its components, CBVD and HD in both sexes. The crude rates in men of any age group were almost always larger than those in women, especially regarding EC. An exception to that occurred with HD; however, in these as well as in all others causes, the standardized rates were higher in men than women (Table 1).

The mortality per 100,000 inhabitants for EC was higher in men than women, but the ratio of men to women decreased with the increase in age. The same occurred with IDCD, with the exception of the youngest group, in which the sex ratio was the same as that in the oldest group. In contrast, the sex ratio of the mortality rates for CD, CBVD, and HD increased up to the 50-59 years group, plateaued, and decreased from the 70-79 years group onwards (Table 1).

Table 2 shows that the proportional mortality related to EC dropped sharply in both sexes across the age groups, decreasing from the youngest to the oldest groups. The IDCD rates were relatively stable across the age groups, showing a subtle predominance of older individuals among men and age extremes among women. The proportional mortality related to CD, CBVD, and HD increased up to the age group of 60-69 years in men and 70-79 years in women and then plateaued. An exception occurred with HD rates, which showed the highest proportional mortality in the oldest age group.

The proportional mortality of EC rates showed a sex ratio markedly unfavorable to men (Table 2), especially up to the age group of 60-69 years. The opposite occurred with IDCD rates, which showed a balance between men and women across almost all ages. In the CD, CBVD, and HD rates, the sex ratio was always unfavorable to women, especially up to the 40-49 years group.

**Figure 1:**
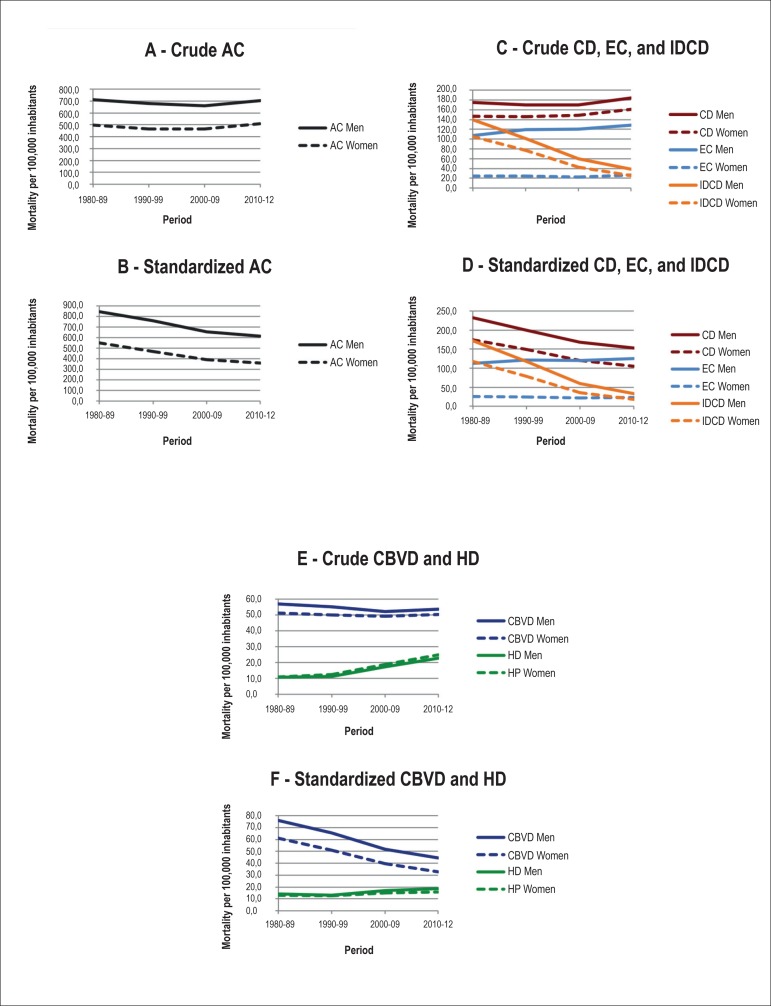
Crude and standardized mortality rates from all causes (AC), circulatory diseases (CD), external causes (EC), Ill-defined causes (IDCD), cerebrovascular diseases (CBVD) and hypertensive diseases (HD), per 100.000 inhabitants, according to sex and observation period - Brazil, 1980-2012.

In Figure 1, the graphs show temporal trends of crude and standardized mortality rates per 100,000 inhabitants according to the causes of death. The crude mortality rates due to AC of death declined until the first decade of the 21st century and increased after that (Figure 1-A). However, when we considered the age of the population, we observed that the decline in standardized rates was constant across the periods, even though it was slightly lower in recent years (Figure 1-B). All crude and standardized mortality rates were higher in men compared with women.

The mortality rates for EC were always higher in men, but showed a subtle trend to an increase in men, compared with relative stability in women. In contrast, the IDCD rates showed a sharp decline in both sexes. These trends were similar for crude and standardized rates by age (Figure 1-C and 1-D).

The crude mortality rates due to CD (Figure 1-C) and two of its components, CBVD and HD (Figure 1-E), showed different trends over the study period. While the CD and CBVD rates decreased up to the first decade of the 21st century and subsequently increased, the crude mortality from HD increased throughout the period, particularly during the transition between the 20th and 21st centuries. After standardization of mortality rates by age, we observed that the rates of CD (Figure 1-D) and CBVD (Figure 1-F) showed a similar decline, which did not occur with the HD rates (Figure 1-F), which increased slightly after the 1990's decade.

## Discussion

In women, the proportional mortality rates due to CD, CBVD, and HD in virtually all ages was higher than those in men, despite a male predominance in the mortality rates per 100,000 inhabitants. This occurred because the EC were more important in men than women, especially among the youngest. The contribution of the IDCD rates to the same effect was more subtle across all age groups.

The predominance of men in the mortality rates due to CD, CBVD, and HD may be associated with the hormonal protection attributed to women.^17^ This is relevant since the hormonal protection seems less sharp in the youngest group and also in the oldest group after the sixth decade of life. Nevertheless, it is necessary to consider other factors, such as environmental ones, especially factors related to working characteristics that distinguish men and women. Still, it is necessary to consider that women expose themselves to diagnostic attention more often making their causes of death easier to be established,^18^ which can explain the lower ratios between sexes in deaths due to HD.

Mortality rates due to CBVD have been decreasing consistently in developed countries.^4,5,19-21^ The same is occurring in Brazil,^9,22^ both in men and women. However, this was not observed in the HD rates, as also encountered by Kung and Xu.^23^ These authors analyzed the deaths related to hypertension between 2000 and 2013 in the United States and observed a 23.1% increase in the standardized mortality rates for HD per 100,000 inhabitants.

Many factors may explain this increase in deaths due to HD. It may reflect an increase in the prevalence of hypertension, from 24.3% in 2012^3^ to an estimated 20% in 2006.^24^ This increase may also be related to a larger coverage of the program Family Health Strategy (*Estratégia de Saúde da Família*), which included around 54 million Brazilians in 2006, allowing more frequent and earlier diagnosis of hypertension.^25^ In addition, the diagnostic criteria for hypertension changed across the observation period of this study, which led individuals with lower blood pressure levels to be diagnosed as new hypertension cases.^26^

Changes may also have occurred in the way the death certificates are completed. When more attention is dedicated to underlying causes of death such as hypertension, the role of these causes when the underlying cause of death is coded in the death certificate cannot be ignored.^27^ Finally, we must mention the growth of the population in terms of number and age, leading to increases in the number of individuals reaching more advanced ages and probabilities of death due to chronic diseases such as HD.^11,25^

Although the focus of this study was to evaluate the rates of CBVD and HD as causes of death, we also had to observe what occurred with the mortality from EC and IDCD. The reason is that the EC, comprising mostly accidents and violence, compete with the other causes of death by removing from the population individuals who could have died due to the former causes. Since these were clearly the most relevant causes in men aged less than 40 years, this group interferes with the analysis of the trends in other causes. This effect was not so relevant in women, who presented a balanced trend in the mortality rates for EC throughout the observation period.

The IDCD are related to morbid states not disclosed in death certificates, often because the deaths occurred in emergency units or without appropriate medical monitoring.^28^ Therefore, they may include undiagnosed cases of CBVD and HD, as observed by Oliveira et al.^29^ However, even if it the IDCD rates include other causes of death this deleterious effect is expected to attenuate over time, since these rates showed a sharp temporal decline.

We did not analyze multiple causes of death in this study since we only considered the underlying cause reported in death certificates. This became a limitation of the study since we are unaware of other causes associated with hypertension and other morbidities that may have also been contributed to the deaths.^27^ In the future, it will be necessary to evaluate how these diseases associated with HD and CBVD evolve over time.

We must also highlight that since this is a study about underlying causes of death, failures in the completion of the death certificates could have interfered with the appropriate coding of the death cause. As observed by Mendonça et al.^30^, one of the main problems with completion of death certificates is that physicians are often unaware of the importance of reporting accurately the sequence of events that culminated with the death.^30^ However, since this represents a generalized problem, potential flaws would affect AC of death and not only the CD or CBVD. We must emphasize the progressive improvement in the completion of death certificates that has been observed in several Brazilian regions, with an increase in the number of lines completed and reduced mortality rates due to IDCD.^27^

## Conclusions

This study described the trends in mortality rates due to CD, CBVD, and HD in Brazil over a recent period of 32 years, and shows the growing importance of HD as a cause of death, in contrast to CBVD and CD. It also shows that the proportional mortality rates due to CD and CBVD in women were higher than those in men, probably due to an unequal sex distribution of EC as causes of death, since these were much more relevant in men. Finally, we showed that there is a need for greater clarification about the participation of HD in deaths. This should stimulate a decrease in hypertension and prevent related deaths, considering that the increase in mortality rates related to this condition cannot be explained based on current knowledge.
